# *PHF6* and *JAK3* mutations cooperate to drive T-cell acute lymphoblastic leukemia progression

**DOI:** 10.1038/s41375-021-01392-1

**Published:** 2021-08-31

**Authors:** Shengnan Yuan, Xiaomin Wang, Shuaibing Hou, Tengxiao Guo, Yanjie Lan, Shuang Yang, Fei Zhao, Juan Gao, Yuxia Wang, Yajing Chu, Jun Shi, Tao Cheng, Weiping Yuan

**Affiliations:** 1 State Key Laboratory of Experimental Hematology, National Clinical Research Center for Blood Diseases, Institute of Hematology and Blood Diseases Hospital, Chinese Academy of Medical Sciences and Peking Union Medical College, Tianjin, China; 2 Department of Neuro-oncology, Cancer Center, Beijing Tiantan Hospital, Capital Medical University, Beijing, China

**Keywords:** Acute lymphocytic leukaemia, Oncogenes, Haematopoietic stem cells

## Abstract

T-cell acute lymphoblastic leukemia (T-ALL) is a malignant hematologic disease caused by gene mutations in T-cell progenitors. As an important epigenetic regulator, *PHF6* mutations frequently coexist with *JAK3* mutations in T-ALL patients. However, the role(s) of *PHF6* mutations in JAK3-driven leukemia remain unclear. Here, the cooperation between JAK3 activation and PHF6 inactivation is examined in leukemia patients and in mice models. We found that the average survival time is shorter in patients with *JAK/STAT* and *PHF6* comutation than that in other patients, suggesting a potential role of PHF6 in leukemia progression. We subsequently found that *Phf6* deficiency promotes *JAK3*^*M511I*^-induced T-ALL progression in mice by inhibiting the Bai1-Mdm2-P53 signaling pathway, which is independent of the JAK3/STAT5 signaling pathway. Furthermore, combination therapy with a JAK3 inhibitor (tofacitinib) and a MDM2 inhibitor (idasanutlin) reduces the *Phf6* KO and *JAK3*^*M511I*^ leukemia burden in vivo. Taken together, our study suggests that combined treatment with JAK3 and MDM2 inhibitors may potentially increase the drug benefit for T-ALL patients with *PHF6* and *JAK3* comutation.

## Introduction

T-cell acute lymphoblastic leukemia (T-ALL) is one of the most common hematologic malignancies, resulted from gene mutations and genomic rearrangements in T-cell progenitors [1, 2]. The 5-year survival rate of adult T-ALL patients is less than 50%, and the mortality rate of relapsed adult T-ALL can be as high as 90% [3]. Recent studies have shown that T-ALL results from multistep transformation processes that involve the accumulation of genetic defects, including activating mutations of *NOTCH1* or *JAK-STAT*, super-enhancer generating mutations of *TAL1*, deep deletion of cell cycle-related genes (such as *CDKN2*, *RB1*, and *CDKN1B*), and inactivating mutations of *WT1*, *LEF1*, *GATA3*, and *PHF6* [4–6]. The close interrelationship between key regulators of early T-cell development and T-ALL oncogenic signals is best illustrated by the prominent roles of the JAK-STAT signal pathway in T-ALL [5, 7, 8]. More than 25% of T-ALL patients carry *JAK-STAT* mutations [9–11], of which *JAK3* mutation is the most frequent in T-ALL cases [8, 9, 12, 13]. The continuous activation of JAK-STAT signaling has been shown to play an essential role in T-ALL initiation and progression [7, 8, 12, 13].

JAK family kinases are nonreceptor tyrosine kinases that function as signal transducers to activate STAT protein to support the differentiation, proliferation and survival of early T-cell progenitors [10]. Activated STAT proteins translocate to nucleus and act as transcription factors to regulate gene expression and/or induce novel epigenetic changes [11]. Aberrant JAK signaling has been linked with T-ALL development. *JAK1* mutations can be found in 10–15% of T-ALL and 1–2% of acute myeloid leukemia (AML) patients [14, 15]. *JAK2*^*V617F*^ mutation is mainly associated with myeloproliferative neoplasms [16]. *JAK3* mutations can be identified in 16.1% of T-ALL cases. *JAK3* plays a key role in regulating T and B cell development [9, 13, 17], while the rate of the most common *JAK3* mutation (M511I) is 34.7% in all *JAK3* mutations [8, 9, 13]. Transplantation of mice with bone marrow (BM) progenitor cells expressing the active *JAK3*^*M511I*^ mutant allele induces a lymphoproliferative disorder followed by a T-ALL-like disease [12]. Moreover, the *JAK3*^*M511I*^ mutation induces phosphorylation and activation of STAT5, which subsequently activates the oncogenes to drive leukemia cell proliferation [8, 12].

It has been reported that *JAK3* mutations could act as “driver” mutations in T-ALL. Interestingly, *JAK3* mutations are frequently accompanied by a high number of genetic changes in T-ALL, such as changes in epigenetic regulatory genes (*ASXL1*, *DNMT3A*, *EED*, *EZH2*, *PHF6*, and *SUZ12*) [5, 13, 17, 18]. *PHF6* is one of the most common mutated epigenetic regulatory gene coexisting with *JAK3* in T-ALL patients [8, 17, 19]. Studies have found that *Phf6*-deficient HSCs had higher proliferation and reconstitution capacity than the wild-type HSCs, although loss of *Phf6* alone is not sufficient to induce aberrant hematopoietic transformation [20–23]. Nevertheless, *Phf6* loss could significantly accelerate leukemia development driven by aberrant expression of *TLX3* or lower the threshold of *NOTCH1*-induced T-ALL development. *Phf6* deficiency could also activate leukemia stem cell transcriptional programs and enhance T-ALL leukemia-initiating cell (LIC) activity [20–22]. While these studies provide mechanistic insight into the importance of PHF6 in regulating early T-cell development, the mechanism of how PHF6 and JAK3 co-occurring events drive leukemogenesis still needs to be functionally dissected and elucidated.

Here we examined the role of cooperatively *PHF6* mutations in the context of *JAK/STAT* mutant-induced leukemia in patients and further functionally dissect their biological events using *JAK3*^*M511I*^ T-ALL mouse model. We found that *PHF6* mutations frequently coexist with *JAK3* mutations in T-ALL patients, and *Phf6* comutation with *JAK3* can drive aggressive leukemia in mice.

## Materials and methods

### Generation of leukemia mice models

*Phf6* conditional deletion mice were generated using the homologous recombination technique and mated with *Vav1-Cre* or *Mx1-Cre* mice to delete *Phf6* in hematopoietic cells. The mice, including donors and recipients used in all our experiments were male mice of 8 weeks. The MSCV-JAK3^M511I^-IRES-GFP retroviral vector was kindly provided by Dr. Jan Cools [8, 12]. Lineage-negative (Lin^−^) cells were transfected with *JAK3*^*M511I*^-GFP virus and transplanted into male mice to establish *Vav1-Cre;Phf6*^*fl/y*^ + *JAK3*^*M511I*^ and *Mx1-Cre;Phf6*^*fl/y*^ + *JAK3*^*M511I*^ mice models. Detailed mouse lines used in this study have been listed in supplementary Table 1 (Table S1). This research was approved by the Institute Ethics Committee and the Institutional Animal Care and Use Committee (IACUC), Institute of Hematology and Blood Diseases Hospital, CAMS/PUMC, KT2020052-EC-1.

### Extreme limiting dilution analysis of LICs

The recipient mice were sublethally irradiated and received 500,000, 50,000, 5000, 500, or 100 GFP^+^ BM cells. The mice were monitored for 4 months. The extreme limiting dilution LIC data were analyzed with ELDA software online [24].

### Inhibitor experiments

For the in vivo experiment, mice were treated through oral gavage with tofacitinib (40 mg/kg per day), idasanutlin (30 mg/kg per day) (MedChemExpress, New Jersey, USA), or vehicle (0.2% dimethylsulfoxide [DMSO]). The mice received medication daily until death. For the in vitro experiment, MOLT-4 cells were treated with tofacitinib (2.0 μM), idasanutlin (0.5 μM) or 0.2% DMSO in culture medium for 48 h and then proliferation and apoptosis were examined.

### RNA-Seq and ChIP-Seq assay

For RNA-Seq analysis of *Phf6* WT/KO + *JAK3*^*M511I*^ cells, 1 µg RNA per sample was used for RNA sample preparations. Transcriptome sequencing was performed on Illumina NovaSeq 6000 platform (Illumina, CA, USA) to a total target depth of 10 million 150 bp paired end reads. Differential expression analysis was performed by DESeq2 R package (1.16.1).

The ChIP assay was performed with the ChIP assay kit (Cell Signaling Technology, Boston, MA, USA) according to manufacturer’s recommendations. Chromatin from cross-linked HA-PHF6-overexpressed K562 or control cells was sheared using an ultrasonicator (Covaris, S220, ABI, New York, USA) to obtain DNA (100–400 bp). Immunoprecipitation was conducted with ChIP-grade HA-tag antibody (Abcam, ab9110, Cambridge, UK), PHF6 antibody (Sigma-Aldrich, HPA001023, Missouri, USA) or normal IgG antibody (Cell Signaling Technology, CST2729, Boston, MA, USA).

RNA-Seq and ChIP-Seq data are available at GEO under accession number GSE159444 and GSE159549.

## Results

### *PHF6* mutation frequently co-exists with *JAK3* mutation in acute leukemia patients

**Fig. 1:**
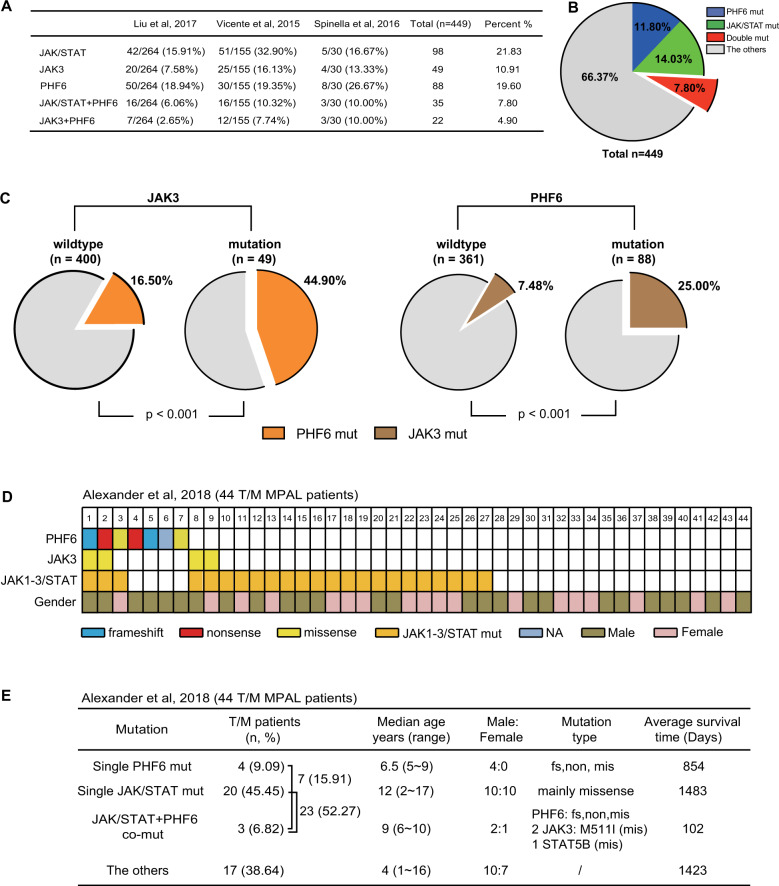
*PHF6* mutations are associated with *JAK3* mutations in AL patients. **A**, **B** The percentage of *JAK3* or *PHF6* mutations in 449 T-ALL patients from three independent clinical centers. **C** Left panel, the frequency of *PHF6* mutations (orange) in T-ALL patients with *JAK3* mutations vs the frequency of *PHF6* mutations in T-ALL patients with WT *JAK3*. Right panel, the frequency of *JAK3* mutations (orange) in T-ALL patients with *PHF6* mutations vs the frequency of *JAK3* mutations in T-ALL patients with WT *PHF6*. Fisher’s exact test was applied to calculate the *P* values to analyze the significance of the positive association between *JAK3* mutations and *PHF6* mutations. **D** Frameshift (fs), nonsense (non), and missense (mis) of *PHF6* or *JAK3* found in 44 T/M MPAL cases from Alexander et al. [19]. Each type of mutation is indicated by a unique color. Gender are also shown for each patient. **E** Total case number, median age, gender ratio, mutation type and average survival time of 44 T/M MPAL patients from Alexander et al. [19].

We analyzed the genetic data of 449 T-ALL cases from different clinical centers, among which *JAK/STAT* mutations occurred in 98 samples (21.83%) [13, 17, 25]. *JAK/STAT* and *PHF6* comutation accounted for 7.80% (35/449) of all T-ALL cases. Interestingly, *JAK3* and *PHF6* comutation accounted for 4.90% of the 449 cases (Fig. 1A, B). *PHF6* mutation occurred in 44.90% of patients with JAK3 mutation (*P* *<* 0.001, Fig. 1C, left). *JAK3* mutation occurred in 25.00% of *PHF6*-mutated patients and was significantly associated with *PHF6* mutation (*P* *<* 0.001, Fig. 1C, right).

We analyzed *PHF6* and *JAK/STAT* mutations in 44 T/myeloid mixed phenotype acute leukemia cases (T/M MPAL) from 102 MPAL patients in Alexander et al. [19]. We found that *PHF6* and *JAK/STAT* comutation accounted for 6.82% (3/44) in total cases (Fig. 1D, E and Table S2). We assigned the 44 T/myeloid MPAL patients into four groups including *PHF6* and *JAK/STAT* comutation, single *JAK/STAT* or *PHF6* mutation, and non-*PHF6/JAK/STAT* mutation (others), and found that the survival time of the *PHF6* and *JAK/STAT* comutation group was significantly shorter than the single *JAK/STAT* mutation group (*P* *<* 0.0001) or the non-*PHF6/JAK/STAT* mutation group (*P* *<* 0.0001, Figs. 1E and S1A). The combined clinical data suggested that *PHF6* mutations may play a synergetic role with *JAK/STAT* mutations in leukemia development.

### *PHF6* mutation acts as an early event to accelerate *JAK3*^*M511I*^ hematopoietic progenitor cell transformation

**Fig. 2:**
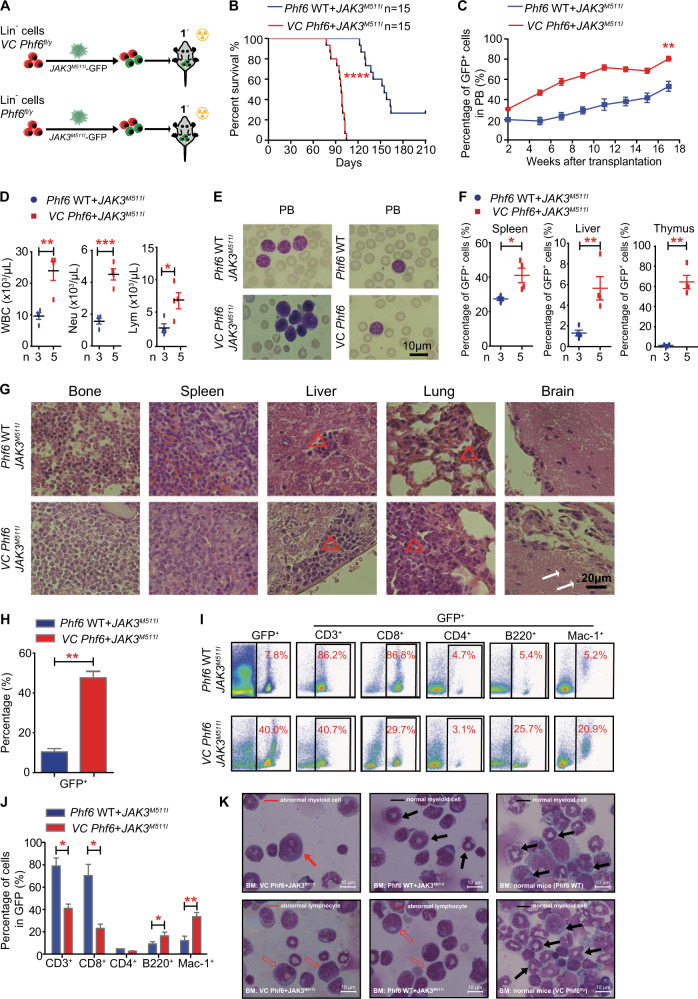
*Phf6* deletion and *JAK3*^*M511I*^ overexpression lead to rapid hematopoietic progenitor transformation to leukemia cells. **A** Scheme of constructing *VC Phf6* + *JAK3*^*M511I*^ and *Phf6* WT + *JAK3*^*M511I*^ T-ALL mouse models. **B** Kaplan–Meier survival curves of *VC Phf6* + *JAK3*^*M511I*^ T-ALL mice and *Phf6* WT + *JAK3*^*M511I*^ T-ALL mice (log-rank test *P* < 0.0001, *n* = 15 per group). *VC Phf6* + *JAK3*^*M511I*^ T-ALL mice (median survival time = 85 days), *Phf6* WT + *JAK3*^*M511I*^ T-ALL mice (median survival time = 143 days). **C** Percentage of GFP^+^ leukemia cells in PB at different time points. **D** The counts of WBCs, lymphocytes (Lym), and neutrophils (Neu) in PB by routine blood tests. **E** Wright–Giemsa staining of peripheral blood cells. **F** Percentage of GFP^+^ cells in spleen, liver, and thymus. **G** Hematoxylin and eosin (HE) staining of BM, spleen, liver, lung, and brain. A red triangle or white arrow indicates the leukemia infiltration area. **H** Percentage of GFP^+^ leukemia cells in BM at 21 weeks after transplantation. **I**, **J** Percentages of T cells, B cells, and myeloid cells in the GFP^+^ population in BM at 21 weeks after transplantation. **K** Wright–Giemsa staining of BM cells.

To evaluate the potential role of *PHF6* mutation in leukemia-initiating events, we generated *Phf6* knockout (*Vav1-Cre;Phf6*^*fl/y*^) and wild-type (*Phf6*^*fl/y*^) mice. We sorted Lin^−^ cells from BM of *Vav1-Cre;Phf6*^*fl/y*^ or *Phf6*^*fl/y*^ male mice and transfected with *JAK3*^*M511I*^-GFP virus. Equal number of GFP^+^ cells were transplanted into male recipients to establish *Vav1-Cre;Phf6*^*fl/y*^ + *JAK3*^*M511I*^ (presented as *VC Phf6* + *JAK3*^*M511I*^) and *Phf6*^*fl/y*^ + *JAK3*^*M511I*^ (as *Phf6* WT + *JAK3*^*M511I*^) mouse lines (Figs. 2A and S1B) [8, 12]. While all mice succumbed to leukemia, the survival time of *VC Phf6* + *JAK3*^*M511I*^ mice was significantly shorter than *Phf6* WT + *JAK3*^*M511I*^ mice (Fig. 2B). The percentage of GFP^+^ leukemia cells was higher in the peripheral blood (PB) of *VC Phf6* + *JAK3*^*M511I*^ mice than *Phf6* WT + *JAK3*^*M511I*^ mice (Figs. 2C and S1C). Although GFP^+^ leukemia cells in the two groups were mainly CD3^+^ or CD8^+^, the percentages of myeloid and B cells were significantly increased in the PB of *VC Phf6* + *JAK3*^*M511I*^ mice when compared with *Phf6* WT + *JAK3*^*M511I*^ mice (Fig. S1D, E). The *VC Phf6* + *JAK3*^*M511I*^ mice showed more aggressive leukemia phenotypes than *Phf6* WT + *JAK3*^*M511I*^ mice, including higher white blood cells (WBCs), neutrophils (Neu), and lymphocytes (Lym) in PB (Figs. 2D, E and S1F). The weights of spleen, liver, and thymus were slightly increased in *VC Phf6* + *JAK3*^*M511I*^ mice when compared with *Phf6* WT + *JAK3*^*M511I*^ mice (Fig. S1G–J). The percentage of GFP^+^ leukemia cells and the degree of extramedullary infiltration in spleen, liver, lung, and brain, were increased in *VC Phf6* + *JAK3*^*M511I*^ mice than *Phf6* WT + *JAK3*^*M511I*^ mice, (Fig. 2F, G). These results demonstrated that *Phf6* deficiency accelerated the initiation of *JAK3*^*M511I*^ hematopoietic progenitor cell transformation.

To further investigate the role of *Phf6* deletion in the transformation of hematopoietic progenitors with *JAK3* mutations, we quantified the biological characteristics of *VC Phf6* *+* *JAK3*^*M511I*^ cells in BM with various surface markers. The percentage of GFP^+^ cells was higher in BM of *VC Phf6* + *JAK3*^*M511I*^ mice (44.1%) than the *Phf6* WT + *JAK3*^*M511I*^ mice (7.8%) (Fig. 2H, I). The percentages of myeloid and B cells in the BM of *VC Phf6* + *JAK3*^*M511I*^ mice were 20.9% and 25.7% respectively, and were higher than the controls (Fig. 2I, J). Wright–Giemsa staining revealed the development of a complex hematolymphoid neoplasm characterized by the coexistence of different populations of atypical cells displaying both lymphoid and myeloid differentiation in *VC Phf6* + *JAK3*^*M511I*^ mice when compared to the controls (Fig. 2K). These results suggested that *Phf6* deficiency induced the polyclonal expansion of *JAK3*^*M511I*^ hematopoietic progenitors.

### *Phf6* deletion promotes *JAK3*^*M511I*^-induced T-ALL progression

**Fig. 3:**
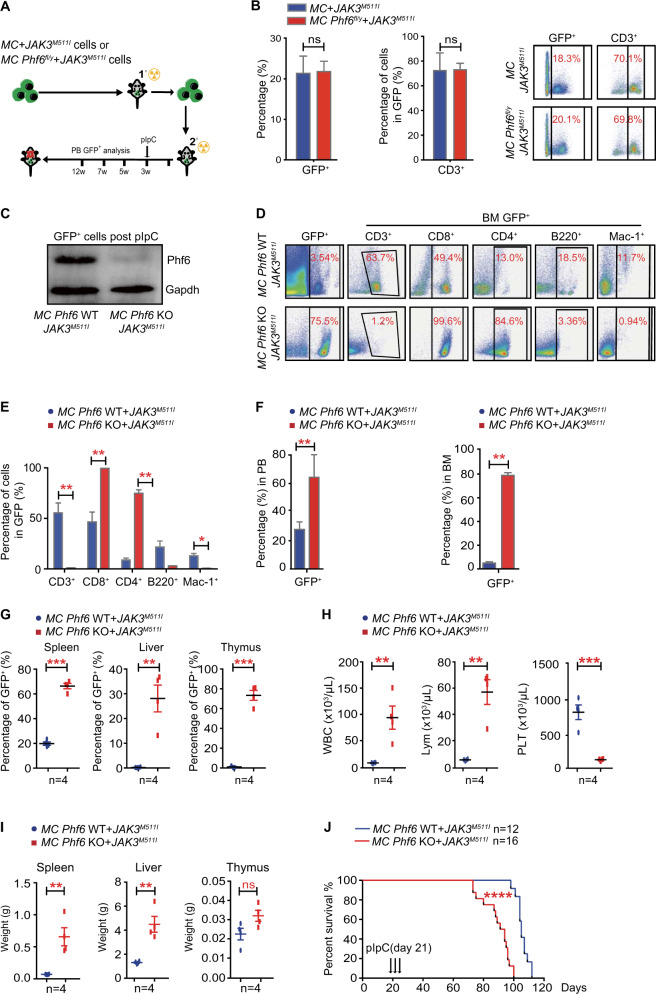
*PHF6* deficiency accelerates T-ALL development in the context of JAK3 mutation. **A** Schematic representation of *Phf6* deletion after *JAK3*^*M511I*^-induced T-ALL in a mouse model. **B** Left panel, percentage of GFP^+^ leukemia cells in the PB of mice at 3 weeks after transplantation. Right panel, percentage of CD3^+^ cells in GFP^+^ leukemia cells in the PB of mice at 3 weeks after transplantation. **C** The Phf6 protein expression level in GFP^+^ cells of T-ALL mice treated with pIpC. **D**, **E** Percentage of T cells, B cells and myeloid cells in GFP^+^ cells in the BM of T-ALL mice treated with pIpC. **F** Percentage of GFP^+^ cells in the PB and BM of T-ALL mice treated with pIpC. **G** Percentage of GFP^+^ cells in the spleen, liver, and thymus of T-ALL mice treated with pIpC. **H** The counts of WBCs, lymphocytes and platelets in the PB of T-ALL mice treated with pIpC by routine blood tests. **I** The weights of the spleen, liver, and thymus of T-ALL mice treated with pIpC. **J** Kaplan–Meier survival curves of *MC Phf6* KO + *JAK3*^*M511I*^ T-ALL mice (*n* = 16) and *MC Phf6* WT + *JAK3*^*M511I*^ T-ALL mice (*n* = 12) after pIpC injection (log-rank test *P* < 0.0001).

Since *JAK3* mutations could act as “driver” mutations in leukemia patients, we investigated whether PHF6 mutations act as subsequent events to promote *JAK3*^*M511I*^-induced leukemia development by generating *Mx1-Cre;Phf6*^*fl/y*^ (*MC Phf6*^*fl/y*^) and *Mx1-Cre;Phf6*^*+/y*^ (*MC*) mice, and constructed *MC Phf6*^*fl/y*^ + *JAK3*^*M511I*^ and *MC* + *JAK3*^*M511I*^ mice (Fig. 3A). *MC Phf6*^*fl/y*^ or *MC* Lin^−^ cells were transfected with retrovirus containing *JAK3*^*M511I*^/GFP ex vivo, and equal number of GFP^+^ cells were injected into lethally irradiated mice. pIpC was injected at 3 weeks post-transplantation to delete *Phf6* when CD3^+^ cells in GFP^+^ cells were above 70% in *JAK3*^*M511I*^-induced T-cell leukemia (Fig. 3A, B). Phf6 deletion was confirmed by western blotting of BM cells from *MC Phf6*^*fl/y*^ + *JAK3*^*M511I*^ and *MC* + *JAK3*^*M511I*^ mice treated with pIpC (presented as *MC Phf6* KO *+* *JAK3*^*M511I*^ or *MC Phf6* WT + *JAK3*^*M511I*^) (Fig. 3C). We sorted GFP^+^ cells from BM of *MC Phf6* WT + *JAK3*^*M511I*^ and *MC Phf6* KO + *JAK3*^*M511I*^ mice, and performed Wright–Giemsa staining to discern the morphological characteristics of GFP^+^ cells. We found that most of the GFP^+^ cells in BM of the two mouse groups were abnormal lymphocytes with larger cell size, irregular nuclear contours, and prominent nucleoli (Fig. S1K). Interestingly, both *MC Phf6* WT + *JAK3*^*M511I*^ and *MC Phf6* KO + *JAK3*^*M511I*^ mice developed CD8^+^ T-ALL without myeloid expansion (Fig. 3D, E). Furthermore, the percentages of GFP^+^ cells in PB, BM, spleen, liver, and thymus were higher in *MC Phf6* KO + *JAK3*^*M511I*^ than in *MC Phf6* WT + *JAK3*^*M511I*^ mice (Fig. 3F, G). The WBC and lymphocyte counts were also increased, while the platelet count was decreased in the PB of *MC Phf6* KO + *JAK3*^*M511I*^ mice (Fig. 3H). The weights of spleens and livers of *MC Phf6* KO + *JAK3*^*M511I*^ were much higher than *MC Phf6* WT + *JAK3*^*M511I*^ mice (Fig. 3I). Importantly, the survival time of *MC Phf6* KO + *JAK3*^*M511I*^ mice was significantly shorter than *MC Phf6* WT + *JAK3*^*M511I*^ mice (Fig. 3J). These data indicated that *Phf6* mutation acquired after *JAK/STAT* mutation could promote *JAK3*^*M511I*^-induced T-ALL progression.

### *Phf6* deficiency increases the activity of leukemia-initiating cells in T-ALL

**Fig. 4:**
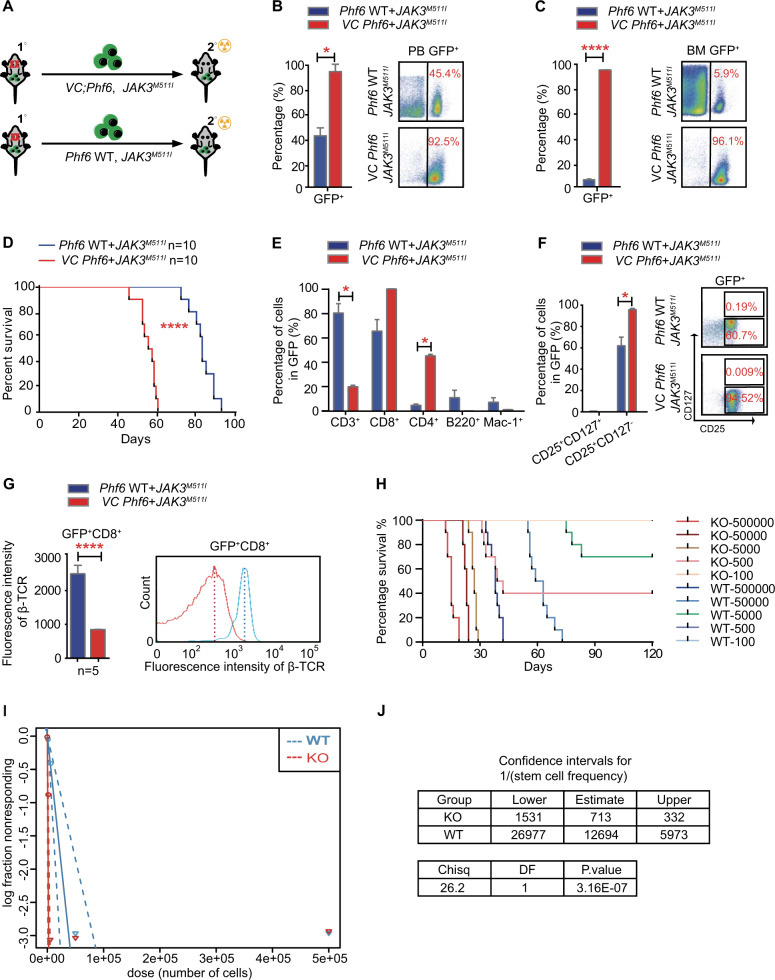
*Phf6* deletion increases the activity of LICs in T-ALL mice. **A** Schematic representation of secondary transplantation. **B**, **C** Percentage of GFP^+^ leukemia cells in the PB and BM of mice at 8 weeks after secondary transplantation. **D** Kaplan–Meier survival curves of *VC Phf6* + *JAK3*^*M511I*^ T-ALL mice and *Phf6* WT + *JAK3*^*M511I*^ T-ALL mice in the secondary transplantation assay (log-rank test *P* < 0.0001, *n* = 10 per group). **E** Percentage of T cells, B cells and myeloid cells in GFP^+^ leukemia cells in the BM of mice at 8 weeks after secondary transplantation. **F** Percentage of GFP^+^ CD25^+^ cells in GFP^+^ leukemia cells in the BM of *Phf6* KO + *JAK3*^*M511I*^ mice and *Phf6* WT + *JAK3*^*M511I*^ mice. **G** The expression of TCR-β in GFP^+^CD8^+^cells of *Phf6* KO + *JAK3*^*M511I*^ mice and *Phf6* WT + *JAK3*^*M511I*^ mice. **H**, **I** *Phf6* KO + *JAK3*^*M511I*^ mice had more LICs (*P* < 0.0001) according to the extreme limiting dilution assay (*n* = 10 per dose). **J** LIC analysis in mice transplanted with *Phf6* knockout and wild-type leukemia cells. Confidence intervals showing 1/ (stem cell frequency) based on **H**.

To determine whether *Phf6* deficiency promotes leukemia initiation by increasing LIC number or activity, GFP^+^ primary *Phf6* WT + *JAK3*^*M511I*^ and *VC Phf6* + *JAK3*^*M511I*^ cells were sorted and transplanted into secondary recipients (Fig. 4A). The mice transplanted with *VC Phf6* + *JAK3*^*M511I*^ cells had more leukemic cells in the PB, BM and extramedullary organs than the controls (Figs. 4B, C and S2A), with significantly shorter survival time than the controls (Fig. 4D). Importantly, secondary leukemia cells arising from *VC Phf6* + *JAK3*^*M511I*^ primary cells in the BM were mainly lymphoid cells (96.3%) (Figs. 4E and S2B), in contrast to the primary *VC Phf6* + *JAK3*^*M511I*^ cells showed much greater heterogeneity (Fig. 2I). Wright–Giemsa staining showed that most GFP^+^ cells of the two groups were abnormal lymphocytes with typical lymphoblastic characteristics (Fig. S2C). These results suggested that the GFP^+^ cells were mainly T leukemic cells based on immunophenotypes and morphological characteristics and the GFP^+^ CD8^+^ T-cell subclone had a dominant growth advantage in *VC Phf6* + *JAK3*^*M511I*^ cell populations.

To further identify the properties of *VC Phf6* + *JAK3*^*M511I*^ leukemia cells, we analyzed T-cell surface markers of GFP^+^ cells (CD4, CD8, CD25, and CD127). The CD25 marker is highly expressed in T cells during embryonic development [26], and we found that the percentage of GFP^+^ CD25^+^ cells was increased in *VC Phf6* + *JAK3*^*M511I*^ mice than the controls, while no obvious CD127 expression was observed (Fig. 4F). Notably, the expression of TCR-β was significantly decreased in *Phf6*-deficient leukemia cells, suggesting that *VC Phf6* + *JAK3*^*M511I*^ cells were more immature (Fig. 4G). Consistent with the presence of more immature T cells in the *VC Phf6* + *JAK3*^*M511I*^ populations, extreme limiting dilution assay demonstrated a marked increase in LICs activity in *Phf6* KO T-ALL cells when compared with *Phf6* WT T-ALL cells, indicating that loss of *Phf6* promoted LICs self-renewal and proliferation (Figs. 4H–J and S2D).

### Loss of *Phf6* enhances leukemia cell proliferation through acceleration of the cell cycle

**Fig. 5:**
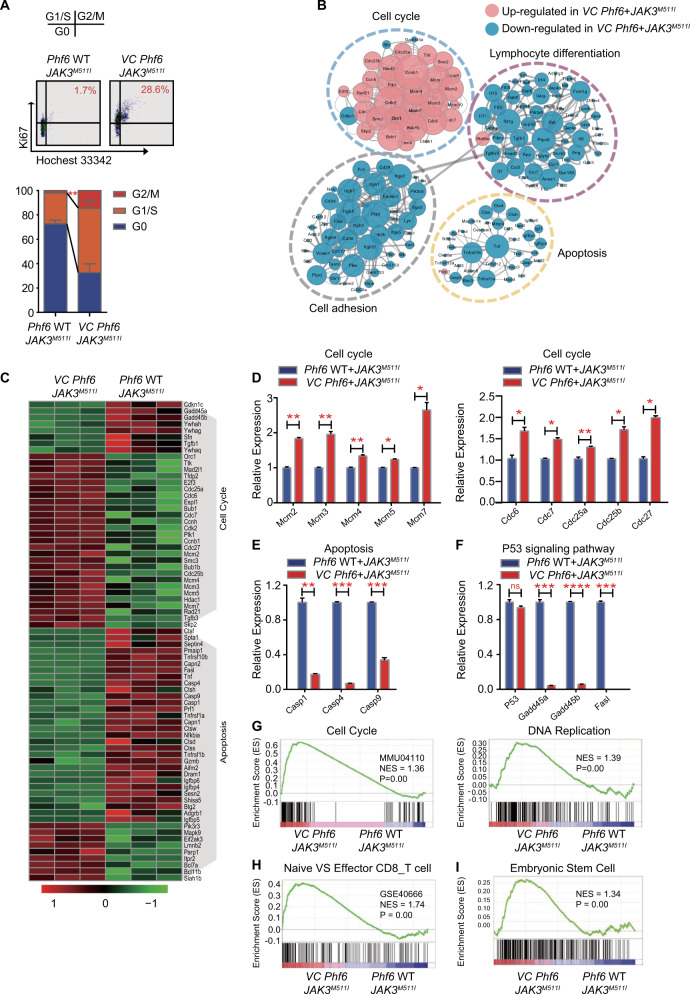
*Phf6* loss accelerates the *JAK3*^*M511I*^ T-ALL cell cycle transcriptional profile. **A** Representative FACS plots showing the cell cycle of GFP^+^ cells in BM (upper panel). Percentage of GFP^+^ cells at each cell cycle stage (lower panel). **B** Gene interaction analysis showing the significantly altered expression pattern in genes that regulate the cell cycle, lymphocyte differentiation, cell adhesion and apoptosis in *VC Phf6* + *JAK3*^*M511I*^ T-ALL cells compared with *Phf6* WT + *JAK3*^*M511I*^ T-ALL cells. **C** Heatmap of cell cycle-related and apoptosis-related genes. **D** Validation of the expression of cell cycle-related genes in GFP^+^ T-ALL cells. **E** Validation of the expression of apoptosis-related genes in GFP^+^ T-ALL cells. **F** Validation of the expression of P53 signaling pathway-targeted genes in GFP^+^ T-ALL cells. **G**–**I** Gene set enrichment analysis (GSEA) of *VC Phf6* + *JAK3*^*M511I*^ T-ALL cells versus *Phf6* WT + *JAK3*^*M511I*^ T-ALL cells.

To identify the definitive role of *Phf6* deficiency in leukemia cell over-proliferation, we investigated cell cycle and apoptosis in *VC Phf6* + *JAK3*^*M511I*^ cells. We found more *VC Phf6* + *JAK3*^*M511I*^ cells were in G1/S and G2/M stages than the control cells (Fig. 5A). However, the cell apoptosis rate was similar in both groups in vivo (Fig. S3A).

To explore the underlying molecular mechanisms of *Phf6* loss in accelerating T-ALL cell proliferation, we analyzed the transcriptional profiles of *VC Phf6* + *JAK3*^*M511I*^ and *Phf6* WT + *JAK3*^*M511I*^ T-ALL cells. It revealed a distinct gene expression signature in *VC Phf6* + *JAK3*^*M511I*^ cells (2377 genes upregulated and 3751 genes downregulated; *P* < 0.05) (Fig. S3B) and these differentially expressed genes were significantly enriched for activities associated with cell cycle, apoptosis, adhesion and lymphocyte differentiation (Fig. 5B). We identified 35 cell cycle-related genes were upregulated, and 39 apoptosis-related genes were downregulated (*P* < 0.05) (Fig. 5C). We further validated that members of Mcm and Cdc gene families, considered to enhance cell proliferation, were upregulated, while genes related to apoptosis, such as Caspase family genes, were downregulated in *VC Phf6* + *JAK3*^*M511I*^ cells (Fig. 5D, E). Notably, the analysis showed *Phf6* loss inhibited the P53 signaling pathway, as this was validated by the decreased mRNA expression of Gadd45a, Gadd45b and Fasl in *Phf6* KO T-ALL cells when compared with the control cells (Fig. 5F).

GO and KEGG analysis revealed active nuclear division and cell cycle in *VC Phf6* + *JAK3*^*M511I*^ cells (Fig. S3C, D), while GSEA showed upregulation of cell cycle progression and DNA replication gene expression in *VC Phf6* + *JAK3*^*M511I*^ cells (Fig. 5G). T-cell maturation might be altered since naive CD8^+^ T-cell-related genes were enriched in *VC Phf6* + *JAK3*^*M511I*^ when compared with the controls (Figs. 5H and S3E). Meanwhile, embryonic stem cell related genes were enriched in *VC Phf6* + *JAK3*^*M511I*^ cells (Fig. 5I). These data suggested that *Phf6* loss promoted *JAK3*^*M511I*^ induced T-ALL progression by accelerating the cell cycle and increasing immature T cells.

### *Phf6* deficiency increases Bai1-mediated P53 degradation

**Fig. 6:**
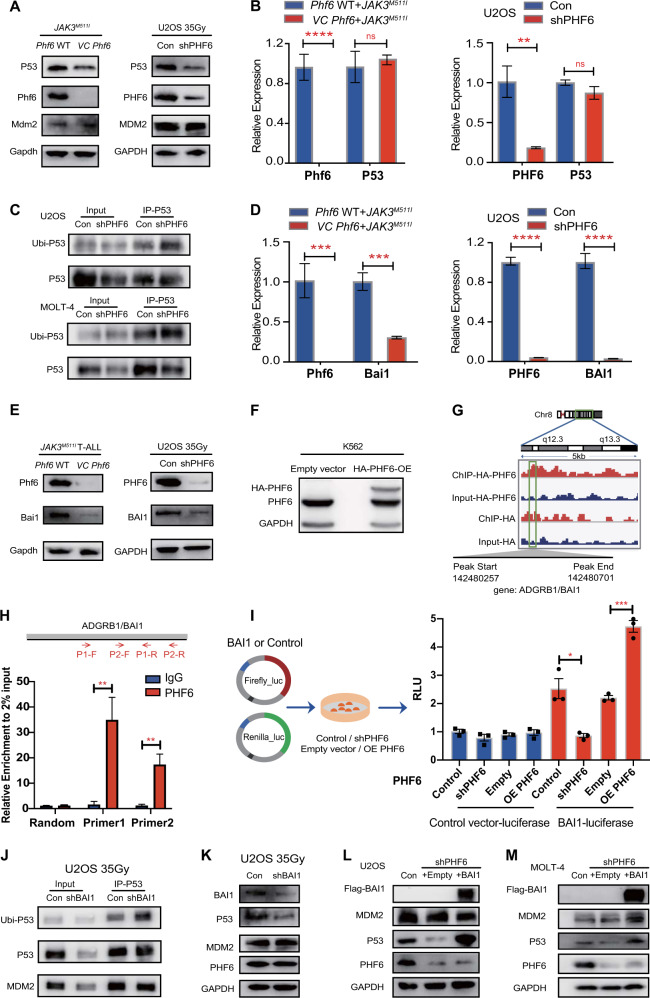
Loss of Phf6 increases P53 degradation by decreasing Bai1 expression. **A** Left panel, the protein expression of P53, Mdm2 and Phf6 in *Phf6* WT + *JAK3*^*M511I*^ and *VC Phf6* + *JAK3*^*M511I*^ leukemia cells. Right panel, the protein levels of P53, MDM2, and PHF6 in PHF6 KD U2OS cells and control cells treated with X-rays (35 Gy). **B** Left panel, the mRNA expression of P53 and Phf6 in *Phf6* WT + *JAK3*^*M511I*^ and *VC Phf6* + *JAK3*^*M511I*^ leukemia cells. Right panel, the mRNA expression of P53 and PHF6 in PHF6 KD U2OS cells and control cells. **C** Co-IP was performed with P53 antibody. P53 ubiquitination was determined by WB with anti-ubiquitin antibody in PHF6 KD/Con U2OS cells treated with X-rays (35 Gy) (upper panel) and PHF6 KD/Con MOLT-4 cells treated with γ-rays (7.5 Gy) (lower panel). **D** Left panel, Bai1 and Phf6 mRNA expression in *Phf6* WT + *JAK3*^*M511I*^ and *VC Phf6* + *JAK3*^*M511I*^ leukemia cells. Right panel, BAI1 and PHF6 mRNA expression in PHF6 KD U2OS cells and control cells. **E** Left panel, protein levels of Phf6 and Bai1 in *Phf6* WT + *JAK3*^*M511I*^ and *VC Phf6* + *JAK3*^*M511I*^ leukemia cells. Right panel, protein levels of PHF6 and BAI1 in PHF6 KD U2OS cells and control cells treated with X-rays (35 Gy). **F** The protein level of HA-PHF6 in PHF6 OE K562 cells. **G** PHF6 binding at the *ADGRB1* (*BAI1*) gene locus in PHF6 OE K562 cells. **H** The relative amount of immunoprecipitated DNA quantified by ChIP-qPCR is given as the percentage of input DNA. **I** Quantification of luciferase activity from K562 cells (PHF6 KD or OE) co-transfected with a luciferase reporter containing the BAI1 sequence (or control sequence) and Renilla luciferase. **J** Co-IP was performed with P53 antibody. P53 ubiquitination was examined in BAI1 KD/Con U2OS cells treated with X-rays (35 Gy). **K** The protein levels of BAI1, P53, MDM2, and PHF6 in BAI1 KD/Con U2OS cells. **L** The protein levels of Flag-BAI1, P53, MDM2, and PHF6 in PHF6 KD + BAI1 OE U2OS cell (lane 3), PHF6 KD + Empty Vector U2OS cell (lane 2) and PHF6 Con U2OS cell (lane 1). **M** The protein levels of Flag-BAI1, P53, MDM2, and PHF6 in PHF6 KD + BAI1 OE MOLT-4 cell (lane 3), PHF6 KD + Empty Vector MOLT-4 cell (lane 2) and PHF6 Con MOLT-4 cell (lane 1).

To determine the underlying molecular mechanism driving the enhanced oncogenic potential of JAK3^M511I^ in the absence of Phf6, we examined the phosphorylation level of Stat5 in *Phf6* WT + *JAK3*^*M511I*^, *VC Phf6* + *JAK3*^*M511I*^, and WT cells. The p-Stat5 was increased in both *Phf6* WT + *JAK3*^*M511I*^ and *VC Phf6* + *JAK3*^*M511I*^ when compared with WT cells, while Phf6 loss did not further enhance p-Stat5 in *VC Phf6* + *JAK3*^*M511I*^ when compared with *Phf6* WT + *JAK3*^*M511I*^ cells (Fig. S4A). We further probed that how Phf6 downregulates P53 signaling pathway, and found that Phf6 deficiency decreased P53 protein expression but not P53 mRNA expression in mouse leukemia cells (Fig. 6A left panel, B left panel). To investigate if PHF6 regulates P53 expression independent of the JAK3-STAT5 signaling pathway, we examined P53 protein levels in PHF6 knockdown (KD) U2OS cells treated with X-ray (35 Gy). We found that the protein expression of P53 was decreased, while the mRNA expression of P53 was unchanged in PHF6 KD U2OS cells when compared with the controls (Fig. 6A right panel, B right panel). The ubiquitination of P53 was significantly increased in PHF6 KD U2OS cells and PHF6 KD MOLT-4 T-ALL cells when compared with the controls (Fig. 6C). These results indicated that PHF6 regulates the ubiquitination of P53.

It has been reported that adhesion G protein-coupled receptor B1 (ADGRB1), also known as brain-specific angiogenesis inhibitor 1 (BAI1), prevents MDM2-mediated P53 ubiquitination, and loss of BAI1 reduces P53 level [27]. We thus examined Bai1/BAI1 expression in *VC Phf6* + *JAK3*^*M511I*^ cells and PHF6 KD U2OS cells, and found that the mRNA and protein expression of Bai1/BAI1 were significantly decreased in both cells in comparison with *Phf6* WT + *JAK3*^*M511I*^ or PHF6 WT U2OS cells, respectively (Fig. 6D, E). Chromatin immunoprecipitation sequencing (ChIP-seq) analysis of HA-PHF6-overexpressing (PHF6 OE) K562 cells (Fig. 6F) showed that PHF6 directly bound to *BAI1* gene (Fig. 6G). We used PHF6 or HA antibody to enrich PHF6 protein in PHF6-OE K562 cells and further verified the binding of PHF6 protein to the *BAI1* DNA sequence by ChIP-qPCR (Figs. 6H and S4B). K562 cells were then co-transfected with a reporter vector containing the *BAI1* sequence (or the control) inserted upstream of firefly luciferase and Renilla luciferase vector. We found that PHF6 KD reduced BAI1 luciferase activity, while PHF6 OE increased BAI1 luciferase activity (Fig. 6I). We further knocked-down BAI1 in U2OS cells and observed that BAI1 KD significantly reduced the P53 level and increased the P53 ubiquitination (Fig. 6j, k). To further investigate whether PHF6 regulates P53 expression via BAI1, the expression of BAI1 was restored in PHF6 KD U2OS cells and PHF6 KD MOLT-4 cells. We found that the expression of P53 was significantly increased in PHF6 KD U2OS cells and PHF6 KD MOLT-4 cells when BAI1 was rescued (Figs. 6L, M and S4C). We further investigated the interaction between PHF6 and BAI1-P53 in the Kasumi-1 cells without irradiation. We found that the mRNA and protein expressions of BAI1 were significantly decreased in PHF6 KD Kasumi-1 cells when compared with that of controls (Fig. S4D, E). The protein level of P53 was also decreased in PHF6 KD Kasumi-1 cells (Fig. S4E) and could be partially rescued when BAI1 was overexpressed in PHF6 KD Kasumi-1 cells (Fig. S4F). Taken together, our data suggested that Phf6 loss inhibited the Bai1-Mdm2-P53 signaling pathway rather than by activating the Jak3/Stat5 signaling pathway.

### Combined treatment with idasanutlin and tofacitinib shows greater antitumor effects in *Phf6* KO + *JAK3*^*M511I*^ T-ALL

**Fig. 7:**
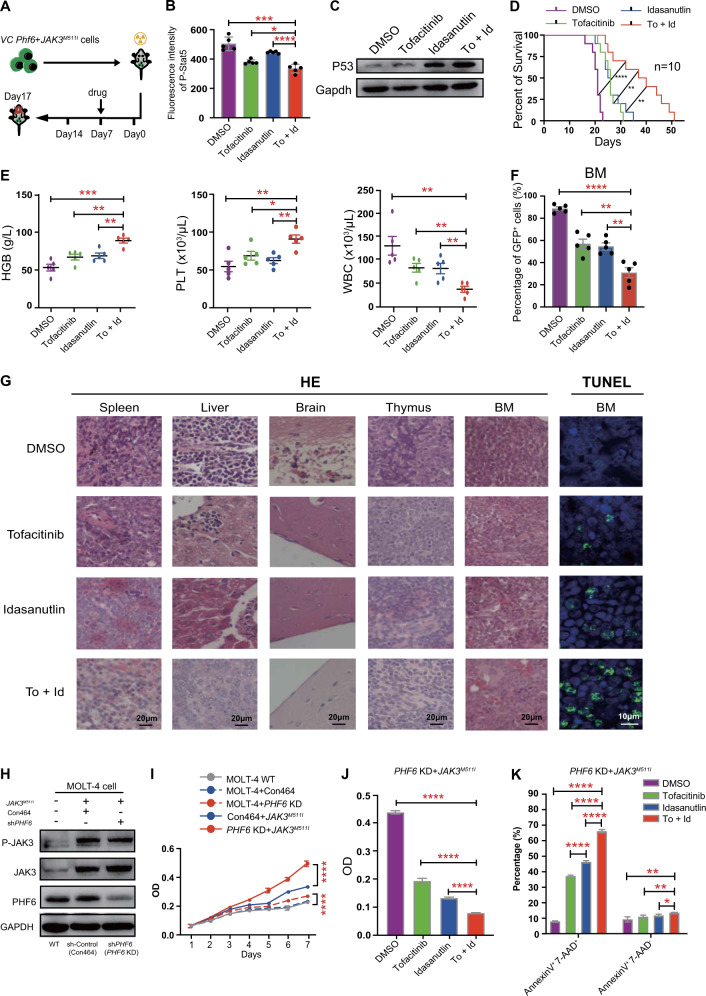
Combined treatment with tofacitinib and idasanutlin prolonged the survival of *Phf6* KO + *JAK3*^*M511I*^ T-ALL mice. **A** Schematic representation of different drug treatments in *VC Phf6* + *JAK3*^*M5111*^ T-ALL mice. **B** Phosphoflow cytometry was used to measure the phosphorylation level of Stat5 in leukemia cells from *VC Phf6* + *JAK3*^*M5111*^ T-ALL mice treated with placebo, single tofacitinib, single idasanutlin, or combined tofacitinib and idasanutlin through oral gavage. **C** Western blotting was used to assess the expression of P53 in leukemia cells from *VC Phf6* + *JAK3*^*M5111*^ T-ALL mice treated with different drugs. **D** Kaplan–Meier survival curves of *VC Phf6* + *JAK3*^*M5111*^ T-ALL mice treated with different drugs (*n* = 10 per group). Mice treated with combined tofacitinib and idasanutlin VS placebo (log-rank test *P* < 0.0001), single tofacitinib (log-rank test *P* = 0.0019) or single idasanutlin (log-rank test *P* = 0.0034). **E** The counts of HGB, PLT, and WBC in PB by routine blood tests. **F** The percentage of GFP^+^ leukemia cells in BM from *VC Phf6* + *JAK3*^*M5111*^ T-ALL mice treated with different drugs. **G** Immunohistochemical staining for hematoxylin and eosin (HE) (magnification, ×40) and TUNEL staining (green represents TUNEL, blue represents DAPI, magnification, ×60) in BM, spleen, liver, brain, and thymus from *VC Phf6* + *JAK3*^*M5111*^ T-ALL mice treated with different drugs. **H** Construction of *PHF6* KD + *JAK3*^*M511I*^ and *PHF6* Con464 + *JAK3*^*M511I*^ MOLT-4 cells and their WB verification. **I** The proliferation of MOLT-4 *PHF6* KD + *JAK3*^*M511I*^ cells was much faster than MOLT-4 *PHF6* Con464 + *JAK3*^*M511I*^, MOLT-4 *PHF6* KD, MOLT-4 Con464 or MOLT-4 WT cells respectively. **J**, **K** Proliferation and apoptosis of MOLT-4 *PHF6* KD + *JAK3*^*M511I*^ cells were examined in vitro with various drug treatment of 0.2% DMSO, single tofacitinib (2.0 μM), single idasanutlin (0.5 μM) or combined tofacitinib + idasanutlin for 48 h.

Since P53 signaling pathway plays a central role in the pathogenesis of *Phf6* KO + *JAK3*^*M511I*^ T-ALL, we sought to determine whether dual activation of P53 and inactivation of JAK3 would be beneficial in *Phf6* KO + *JAK3*^*M511I*^ T-ALL mice. Tofacitinib is a JAK3-specific inhibitor [12]. Idasanutlin is a selective MDM2 antagonist that can activate P53 [28, 29]. *VC Phf6* + *JAK3*^*M511I*^ T-ALL mice were treated with placebo, single tofacitinib, single idasanutlin, or combined tofacitinib + idasanutlin (To + Id) (Fig. 7A). We measured p-Stat5 and P53 in *VC Phf6* + *JAK3*^*M511I*^ BM cells from treated or nontreated mice. We found that p-Stat5 was decreased (Figs. 7B and S5A), and P53 was increased in mice treated with To + Id (Fig. 7C). The survival time of mice treated with To + Id was significantly longer than mice treated with placebo, single tofacitinib or idasanutlin (Fig. 7D). The counts of hemoglobin and PLT were increased, while the count of leukemia cells was decreased in the PB of mice treated with To + Id when compared with mice in other groups (Fig. 7E). Additionally, the percentages of GFP^+^ leukemia cells in the PB, BM, spleen, liver, and thymus were decreased in mice treated with To + Id when compared with other groups (Figs. 7F and S5B–E). The weights of spleen, liver, and thymus were significantly decreased in mice treated with To + Id in comparison with other groups (Fig. S5F, G). HE staining showed that the degree of leukemia cell infiltration in the spleen, liver, brain, thymus, and BM was reduced in mice treated with To + Id when compared with other groups (Fig. 7G). Immunohistochemical staining of Ki67, a marker of cell proliferation, showed significantly reduced staining in the To + Id-treated group when compared with the other groups (Fig. S5H), while TUNEL (TdT-mediated dUTP nick-end labeling) staining showed a significant increase in apoptotic cells in mice treated with To + Id than other groups (Fig. 7G).

Also, we constructed *JAK3*^*M511I*^*-*overexpression MOLT-4 cells that were treated with sh*PHF6* (*PHF6* KD) or sh-Control (*PHF6* Con464) respectively, and confirmed by WB along with MOLT-4 cells (WT) (Fig. 7H). We found that the proliferation of *PHF6* KD + *JAK3*^*M511I*^ cells was much faster than *PHF6* Con464 + *JAK3*^*M511I*^ or other control cells (Fig. 7I). Furthermore, the proliferation of *PHF6* KD + *JAK3*^*M511I*^ MOLT-4 cells was significantly decreased while their percentage of apoptosis was increased after treated with To + Id when compared with single drug or control treatment groups (Fig. 7J, K). Our result thus demonstrated that combination therapy with tofacitinib and idasanutlin can reduce leukemia burden better than single drug treatment in *Phf6* KO + *JAK3*^*M511I*^ mice, which was also validated by the result in human MOLT-4 cells.

## Discussion

Understanding the mechanism of how epigenetic regulatory genes target chromatin and redirect gene transcription and activation in leukemogenesis is imperative for developing novel therapies. As a chromatin remodeling-related gene, *PHF6* is frequently mutated in T-ALL patients [13, 17, 30]. Here, we demonstrated that *PHF6* mutations more commonly coexisted with *JAK/STAT* mutations in T-ALL patients (Fig. 1A). The average survival time of patients with *JAK/STAT* and *PHF6* comutation was shorter than that of patients without this comutation (Figs. 1E and S1A). *Phf6* deletion led to rapid development of *JAK3*^*M511I*^-induced T-ALL by inhibiting the Mdm2-P53 signaling pathway. Leukemia progression can be contained better by specific inhibition of JAK3 and Mdm2-P53 in *Phf6*-deficient and *JAK3*^*M511I*^ T-ALL cells (Fig. 7D). Furthermore, we revealed that the progression of *JAK3*^*M511I*^-induced T-ALL from low to high malignancy is triggered by the coexistence of *PHF6* mutation, and that provided a potential therapeutic window for the modulation of P53 and JAK3 activity in the treatment of T-ALL patients with *PHF6* and *JAK3* comutation.

It has been reported that Phf6 is essential for HSC homeostasis and T-ALL initiation, although Phf6 appears to play a modest role in normal T-cell differentiation and proliferation [20]. Loss o*f Phf6* slightly reduced number of T cells in PB and BM, but did not lead to spontaneous hematological malignant transformation in mice [20]. Earlier studies theorized that PHF6 may play important roles in lineage-specification during leukemogenesis. For example, lack of *Phf6* promoted *Notch*-induced T-ALL initiation and MLL-AF9-induced AML progression, while decelerated the development of BCR-ABL1-induced B-ALL [31]. However, Thomas and colleagues suggested that PHF6 might not act in a strictly lineage-dependent manner. They found that *Phf6* knockout accelerated *TLX1/TLX3*-induced B-ALL [22], and that is different from the oncogenic role of Phf6 in BCR-ABL1-induced B-ALL. It indicated that the role(s) of PHF6 may depend on the combination of oncogenic mutations and specific molecular pathways that drive leukemia. In our studies, we found that primary *Phf6* KO + *JAK3*^*M511I*^ progenitor cells developed a complex hematolymphoid neoplasm characterized by the coexistence of different populations of atypical cells (Fig. 2I, J). However, when we depleted *Phf6* in *MC Phf6* *+* *JAK3*^*M511I*^ mice with pIpC, leukemia cells were mainly lymphoid cells (Fig. 3D, E). These results suggested that if PHF6 mutation and JAK3 mutation occurred in hematopoietic progenitors, it may induce a complex hematolymphoid neoplasm. When PHF6 mutation acted as “a secondary strike” in *JAK3*^*M511I*^-induced T-ALL, it might also promote the T-ALL progression. Based on our observations, we reasoned that PHF6 might function as a tumor suppressor, while PHF6 loss or mutations favors T-ALL initiation by lowering the threshold for subsequent oncogenic transformation in hematopoietic progenitors, and postulated the role in promoting T-ALL development.

**Fig. 8:**
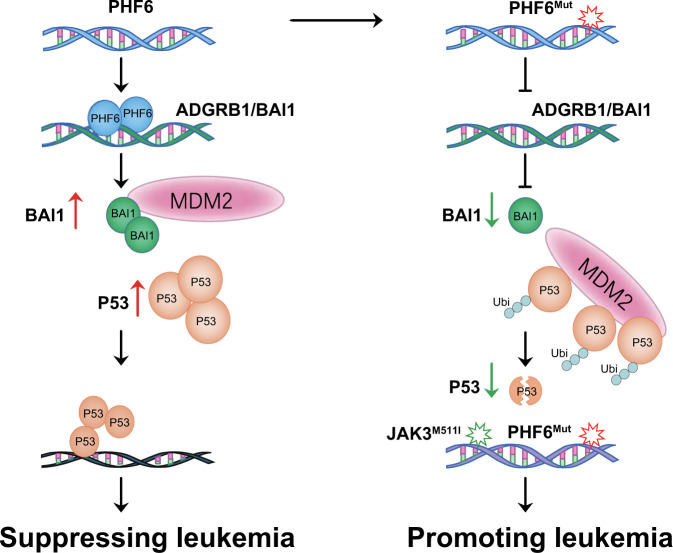
A proposed model for the role of PHF6 in T-ALL initiation by regulating BAI1-MDM2-P53 signaling pathway. In *JAK3*^*M511I*^-induced leukemia, PHF6 could bind to *ADGRB1* (*BAI1*) gene, increase its expression, upregulate BAI1 level, prevent MDM2-mediated P53 ubiquitination, stabilize the P53 protein, and suppress leukemia development (Left panel). In the same leukemia model, loss of PHF6 could downregulate the expression of BAI1, lead to increased MDM2-P53 binding and P53 degradation, and accelerate *JAK3*^*M511I*^-induced T-ALL progression (Right panel).

We further demonstrated how PHF6 functions in *JAK3*^*M511I*^-induced T-ALL independent of the Jak3-Stat5 signaling pathway. As expected, we found that Stat5 was activated in both *Phf6* KO/WT + *JAK3*^*M511I*^ cells (Fig. S4A) [12]. However, the Mdm2-P53 signaling pathway was inhibited only in *Phf6* KO + *JAK3*^*M511I*^ but not in *Phf6* WT + *JAK3*^*M511I*^ leukemia cells (Fig. 6A). This suggested that T-ALL progression due to Phf6 loss is dependent on additional signaling pathways. BAI1 belongs to the adhesion subgroup of GPCRs, which functions at preventing MDM2-mediated P53 ubiquitination, and its loss could substantially reduce the P53 level [27]. Consistent with prior findings showing that P53 loss increased penetrance and accelerated progression of leukemia [28, 32], we showed here that PHF6 loss increased *JAK3*^*M511I*^-induced T-ALL initiation by downregulating BAI1 expression, thereby decreasing BAI1 and MDM2 interaction, and destabilizing P53 (modeled in Fig. 8). Thus, our results defined a PHF6-BAI1-P53 signaling axis and linked it with *JAK3*^*M511I*^-induced T-ALL genesis. It will be interesting to assess whether T-ALL patients with *JAK3* and *PHF6* comutation could further benefit from combined therapy with MDM2 and JAK3 inhibitors.

In conclusion, we revealed that *PHF6* and *JAK3* mutations cooperatively drive T-ALL progression probably via inhibiting the BAI1-MDM2-P53 signaling pathway, in addition to activating the JAK3/STAT5 signaling pathway. We further demonstrated that combination therapy with tofacitinib and idasanutlin reduced the *Phf6* KO + *JAK3*^*M511I*^ leukemia burden in vivo. Our study suggested that the combined usage of JAK3 and MDM2 inhibitors should increase the drug benefit for T-ALL patients with *PHF6* and *JAK3* comutation.

## Supplementary information

Supplementary-PHF6
