# Supplementary material for: PHF6 and JAK3 mutations cooperate to drive T-cell acute lymphoblastic leukemia progression
Source: Leukemia. 2021 Aug 31;36(2):370–82. doi: 10.1038/s41375-021-01392-1 (PMC8807395; doi:10.1038/s41375-021-01392-1)
Supplement: Supplementary file 1 — Supplementary-PHF6 [file 41375_2021_1392_MOESM1_ESM.docx]

Supplementary Data for Shengnan Yuan et al.: ***PHF6 and JAK3 mutations cooperate to drive T-cell acute lymphoblastic leukemia progression*** (Including Supplementary Methods, 5 Supplementary Figures and 2 Supplementary tables)

**Supplementary methods**

**Generation of *Phf6* conditional knockout mice**

*Phf6* conditional deletion mice were generated using the homologous recombination technique to flank exon 4 to exon 5 of *Phf6* with two LoxP sequences (Supplementary Fig. 1B). The mice were then mated with *Vav1-Cre* transgenic mice expressing Cre recombinase under the control of the *Vav1* promoter to delete *Phf6* in hematopoietic cells at the embryonic stage. As expected, half of the offspring were *Phf6* wild-type (*Phf6^fl/y^* and *Phf6^fl/fl^*), and the other half were *Phf6* knockout mice (*Vav1-Cre;Phf6^fl/y^* and *Vav1-Cre;Phf6^fl/fl^*). The mice were mated with *Mx1-Cre* transgenic mice to generated *Mx1-Cre;Phf6^fl/y^* (*MC Phf6^fl/y^*) and *Mx1-Cre;Phf6^+/y^* (*MC*) mice. We sorted bone marrow lineage-negative (Lin^-^) cells from male doner mice of 8 weeks and transfected with *JAK3^M511I^*-GFP^+^ virus. We transplanted equal amount of GFP positive cells into recipient mice through tail vein injection to generated *Vav1-Cre;Phf6^fl/y^*+*JAK3^M511I^* (presented as *VC* *Phf6*+*JAK3^M511I^* ), *Phf6^fl/y^*+*JAK3^M511I^* (presented as *Phf6* WT+*JAK3^M511I^*), *Mx1-Cre;Phf6^fl/y^* +*JAK3^M511I^* (presented as *MC Phf6^fl/y^*+*JAK3^M511I^* ) and *Mx1-Cre;Phf6^+/y^* +*JAK3^M511I^* (presented as *MC*+*JAK3^M511I^* ) mice models. All male recipient mice used in our experiments were 8 weeks old. In the *Vav1-Cre* system, mice in primary transplant experiment were analyzed for phenotypes at 21st week post-transplantation. Secondary transplantation using GFP positive cells from primary transplantation was carried out, and mice were examined for phenotypes at 8th week post-transplantation; In the *Mx1-Cre* system, mice were injected with pIpC at third weeks post-transplantation. Phf6 deletion was confirmed by Western blotting analysis of BM cells from *MC Phf6^fl/y^*+*JAK3^M511I^* or *MC*+*JAK3^M511I^* mice treated with pIpC (presented as *MC* *Phf6* KO*+JAK3^M511I^* and *MC Phf6* WT+*JAK3^M511I^,* respectively) and the phenotypic experiments were done at 13th week post-transplant. Animals were housed in the specific pathogen-free (SPF) animal facility of the State Key Laboratory of Experimental Hematology (SKLEH), Institute of Hematology and Blood Disease Hospital. All efforts were made to minimize the suffering of the mice.

**Murine bone marrow transplantation**

Hematopoietic stem and progenitor cells were enriched from the bone marrow of mice and then infected with concentrated retroviral supernatants. After 72 hours, cells were washed in phosphate-buffered saline and injected (1×10^6^ GFP^+^ cells/0.3 mL) into the lateral tail vein of lethally irradiated (9.5 Gy) male recipient mice for further experiments. Same batches of mice were randomly assigned to control and experiment groups. Mice were housed in individually ventilated cages and monitored daily.

**Flow cytometry analyses**

Single-cell suspensions were prepared from peripheral blood, bone marrow, spleen, thymus, liver or lung. The CD3 marker detected in our experiment was a surface expressed CD3. Cells were analyzed using a FACS Canto flow cytometer (BD Biosciences, New Jersey, USA). Data were analyzed with FlowJo software (Tree Star).

**Cell lines and culture**

Cell lines used in our experiment were obtained from the experimental pathology cell bank of the State Key Laboratory of Experimental Hematology (SKLEH) from Institute of Hematology and Blood Diseases Hospital (IHBD). MOLT-4 cell, Kasumi-1 cell, and K562 cell were cultured at 37°C with 5% CO2 in RPMI-1640 medium (Gibco, CA, USA) supplemented with 10% FBS. HEK293T, U2OS cell was cultured in DMEM basic medium with 10% FBS. Experiments were performed within four weeks after fresh viable cells were thawed. U2OS cells used in the study for WB and IP experiments were treated with X-ray (35 Gy), and MOLT-4 T-ALL cells were treated with γ-ray irradiation (7.5Gy).

**Cell cycle analysis**

GFP^+^ cells were sorted from bone marrow. After staining of surface antigens, cells were fixed using the Cytofix Fixation/Permeabilization Kit (BD Biosciences, New Jersey, USA) according to the manufacturer’s instructions. Cells were stained with Ki67 antibody at room temperature for 30 min. Prior to analysis, cells were incubated with 1.62 µM Hoechst 33342 (Invitrogen, Carlsbad, CA) at room temperature. Flow data acquisition was performed using a BD LSRII™ (BD Biosciences, New Jersey, USA) flow cytometry analyzer.

**RNA extraction and quantitative real-time PCR**

RNA from 10^6^ cells was extracted using a RNeasy Mini Kit (Qiagen, Toronto, ON, Canada). One microgram of total RNA was reverse transcribed (RT) with oligo(dT) primers and Super Script III enzyme (Life Technologies, Burlington, ON, Canada). qPCR for ChIP-DNA or RT samples was prepared using SYBR Green Advantage qPCR premix (Clontech, Mountain View, CA, USA), and the results were analyzed on an Mx3000P system (Agilent Technologies Canada, Inc., Mississauga, ON, Canada).

**Co-Immunoprecipitation**

Cells were rinsed with ice-cold PBS and solubilized for 15 min on ice in lysis buffer (1% NP40, 10% glycerol, 135 mM NaCl, 20 mM 1 M Tris-HCl, pH 8.0) supplemented with protease inhibitor. Lysates were then centrifuged in a microfuge at 12,000 rpm for 10 min, and the supernatants were immunoprecipitated at 4°C with antibodies (5 μg/ml) for 2 hours followed by protein A/G magnetic beads (Invitrogen, Carlsbad, CA) overnight. Bound proteins were eluted with protein loading buffer and boiled at 100℃. Antibodies used in the Co-IP/WB assay were as follows: anti-PHF6 antibody (Abcam, ab173304, Cambridge, UK), anti-MDM2 antibody (Abcam, ab16895, Cambridge, UK), anti-P53 antibody (Abcam, ab16465 and ab131442, Cambridge, UK), anti-BAI1 antibody (Novus, NB110-81586, Colorado, USA), anti-ubiquitin antibody (Abcam, ab134953, Cambridge, UK), and anti-GAPDH antibody (Cell Signaling Technology, CST2118, Boston, MA, USA).

**Western blotting analysis**

Western blot analysis was performed using standard protocols. In brief, proteins resolved by SDS-PAGE were transferred to nitrocellulose membranes. Nitrocellulose blots were incubated at room temperature for 1 hour in blocking buffer (TBST with 5% milk), followed by incubation with the indicated antibodies at 4°C overnight. After three 15-min washes with TBST, the blots were incubated with horseradish peroxidase-conjugated secondary antibody (Cell Signaling Technology, Boston, MA, USA). Immunoreactive bands were visualized using enhanced chemiluminescence substrate (Bio-Rad, Hercules, CA, USA).

**Dual-luciferase reporter assay**

Human ADGRB1 (BAI1) DNA sequence or control sequence was inserted into firefly luciferase vector. PHF6 OE or PHF6 KD (knock down) K562 cells were co-transfected with firefly luciferase vector (200 ng) and Renilla (4 ng) luciferase vector (Promega, Madison, WI, USA) using Lipofectamine 2000 (Invitrogen, Carlsbad, CA, USA) and cultured in 96 wells. The Dual Luciferase Reporter Assay System (Promega, Madison, WI, USA) was applied to measure the luciferase activities at 48 h after transfection. Firefly luciferase activities were normalized to Renilla luciferase values as a control and shown as an average of triplicates.

**Immunohistochemistry (IHC)**

The IHC analysis was performed in paraffin-embedded sections using specific antibodies against Ki67 (Cell Signaling Technology, Boston, MA, USA). These slides were then subjected to horseradish peroxidase-linked secondary antibodies for 1 hour at room temperature. Staining was visualized by the DAB substrate kit (Gene Tech, Shanghai, China). Representative IHC images were captured at 40X magnification.

**TUNEL staining assay**

TdT-mediated dUTP nick-end labeling (TUNEL) staining was performed on paraffin-embedded sections using a TUNEL staining kit (Beyotime, Shanghai, China). After treatment with protease K and 0.1% Triton X-100, 50 µL of TUNEL reaction mixture was added to each slide. The samples were incubated in the dark in a humidified chamber for 1 hour at 37°C. Representative images were captured at 60X magnification using a fluorescence microscope.

**Statistical analysis**

All experiments were repeated two to three times with the indicated numbers. Sample size was determined according to experience or the previously published papers. The investigator was not blinded during experiment or assessing the outcome. Distribution was tested using the modified Shapiro–Wilks method. When parameters followed Gaussian distribution, Student’s t test was used for two groups’ analyses and one-way ANOVA was used for comparing more than two groups to evaluate the statistical significance. Data were expressed as mean ± SD. *P* < 0.05 was considered statistically significant. We have compared the similar variance between the groups statistically. Data were processed in GraphPad Prism 8.4.0 software (La Jolla, CA, USA). Kaplan-Meier curves were used to plot survival, and significant differences were calculated with the log rank test.

**Supplementary Figure 1**

**
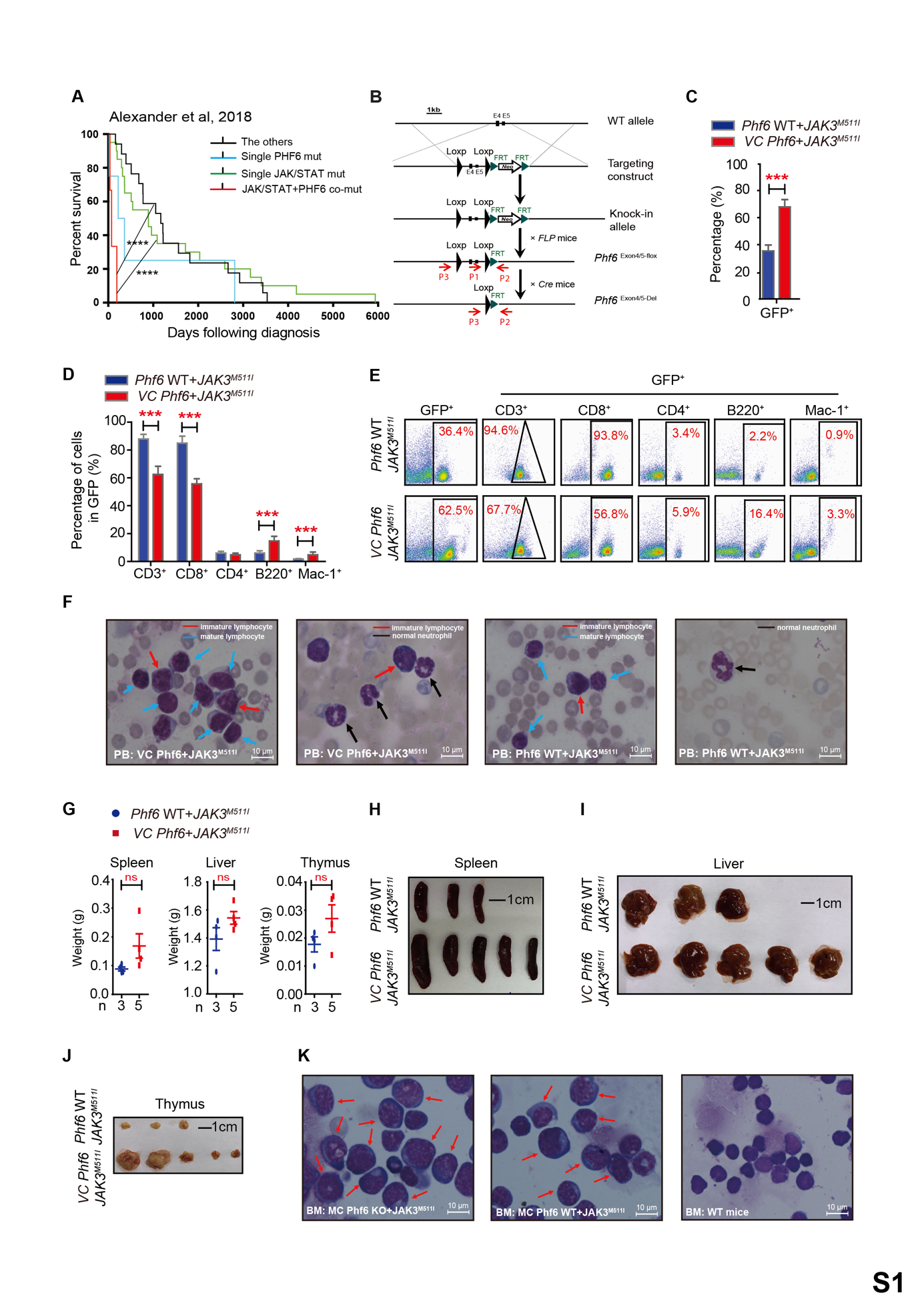
**

**Supplementary Figure 1. *PHF6* deficiency lowers the threshold for oncogenic transformation in hematopoietic progenitors.**

(A) T/M MPAL patients with *JAK/STAT* and *PHF6* comutation had shorter survival time than patients with single *JAK/STAT* mutation (log-rank test *P* *< 0.0001*), single *PHF6* mutation (log-rank test *P* = 0.139) or neither mutation (others) (log-rank test *P* *< 0.0001*) from *Alexander et al*, 2018 [1]. (B) Phf6 knockout mouse model construction strategy. (C) Percentage of GFP^+^ leukemia cells in the PB of mice at 21 weeks after transplantation. (D-E) Percentage of T, B and myeloid cells in the GFP^+^ population in the PB of mice at 21weeks after transplantation. (F) Wright-Giemsa staining of PB cells from *VC* *Phf6+JAK3^M511I^* and *Phf6* WT*+JAK3^M511I^* mice. (G-J) The spleen, liver and thymus were larger in *VC* *Phf6+JAK3^M511I^* mice than in *Phf6* WT*+JAK3^M511I^* mice. (K) Wright-Giemsa staining of GFP^+^ cells in BM of *MC Phf6* KO+*JAK3^M511I^*, *MC Phf6* WT+*JAK3^M511I^* and WT mice.

**Supplementary Figure 2**


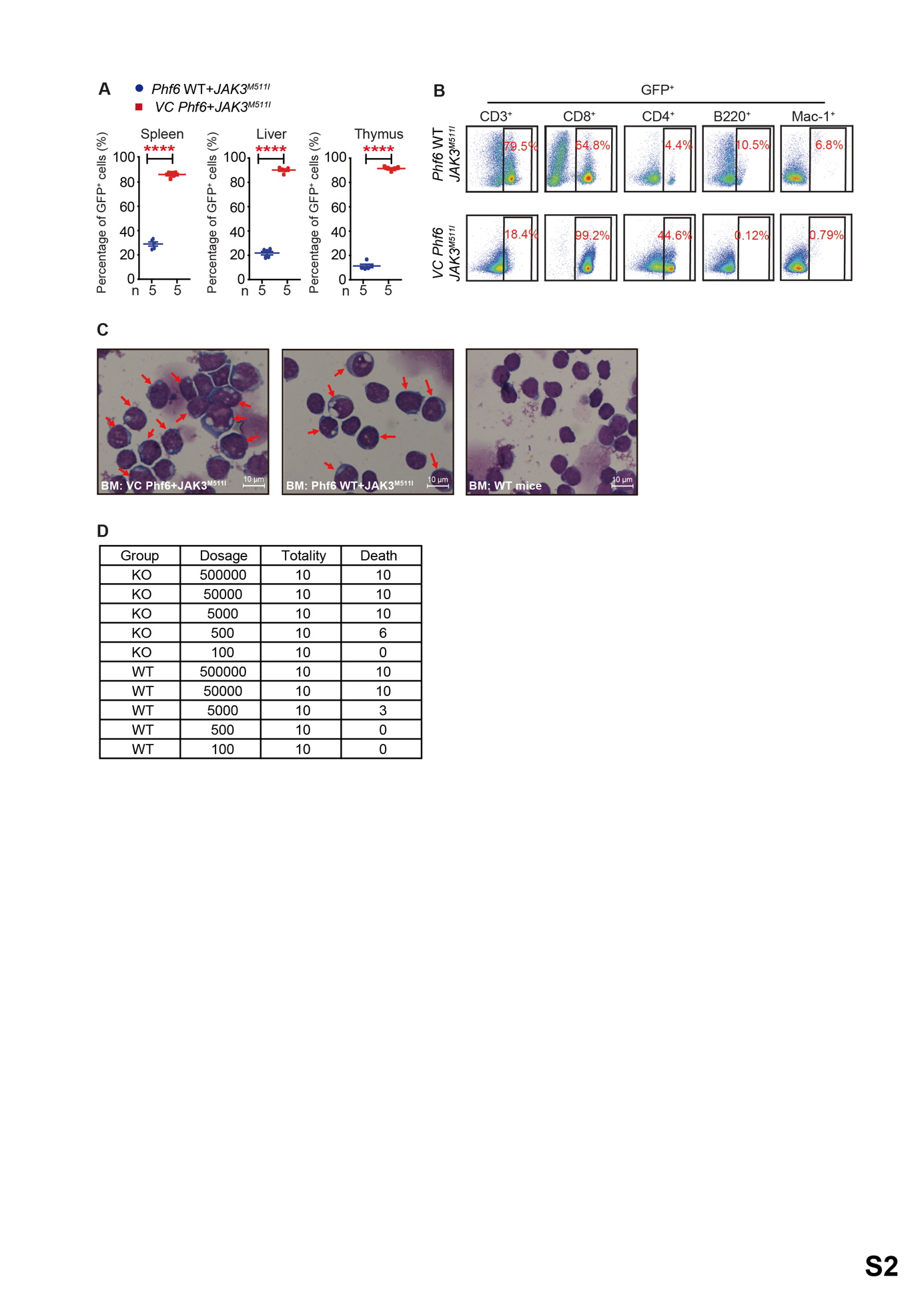


**Supplementary Figure 2. Loss of *Phf6* increases the activity of leukemia initiating cells in T-ALL.**

(A) Percentage of GFP^+^ cells in the spleen, liver and thymus of mice in the secondary transplantation assay. (B) Percentage of T, B and myeloid cells in GFP^+^ leukemia cells in the BM of mice at 8 weeks after secondary transplantation. (C) Wright-Giemsa staining of BM cells from *VC* *Phf6+JAK3^M511I^*, *Phf6* WT*+JAK3^M511I^* and WT mice. (D) Statistics of the number of dead mice after transplantation with different amounts of leukemia cells in the extreme limiting dilution assay.

**Supplementary Figure 3
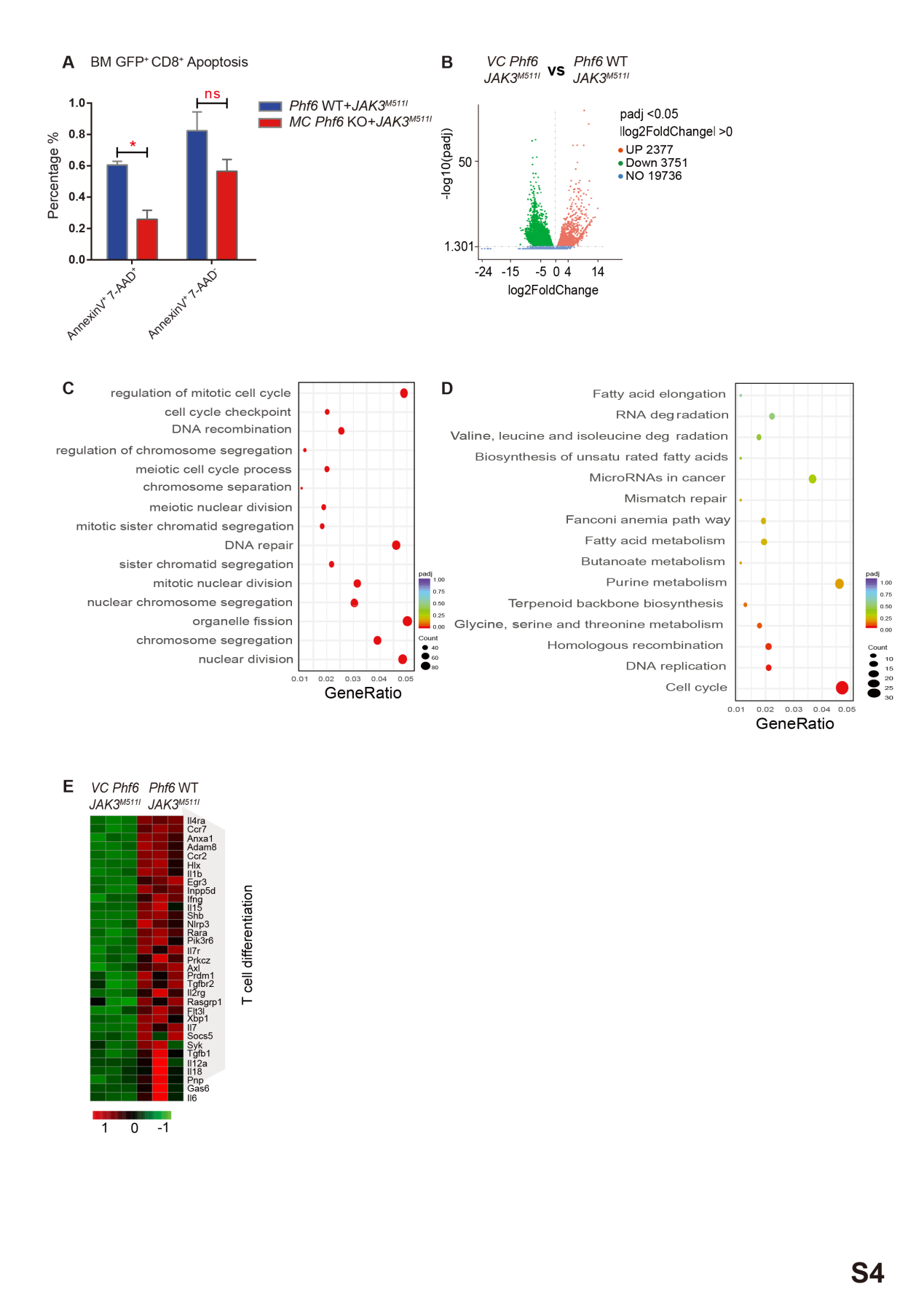
**

**Supplementary Figure 3. *PHF6* deficiency leads to more active transcriptional profiling during leukemia progression.**

(A) The percentage of apoptotic cells in the BM of *VC Phf6*+*JAK3^M511I^* mice and *Phf6* WT+*JAK3^M511I^* mice. (B-E) Bioinformatic analysis of transcriptional profiles in *VC Phf6*+*JAK3^M511I^* and *Phf6* WT+*JAK3^M511I^* leukemia cells: Volcano map of upregulated and downregulated genes (B); GO analysis (C); KEGG analysis (D); heatmap of T cell differentiation-related genes (E).

**Supplementary Figure 4**

**
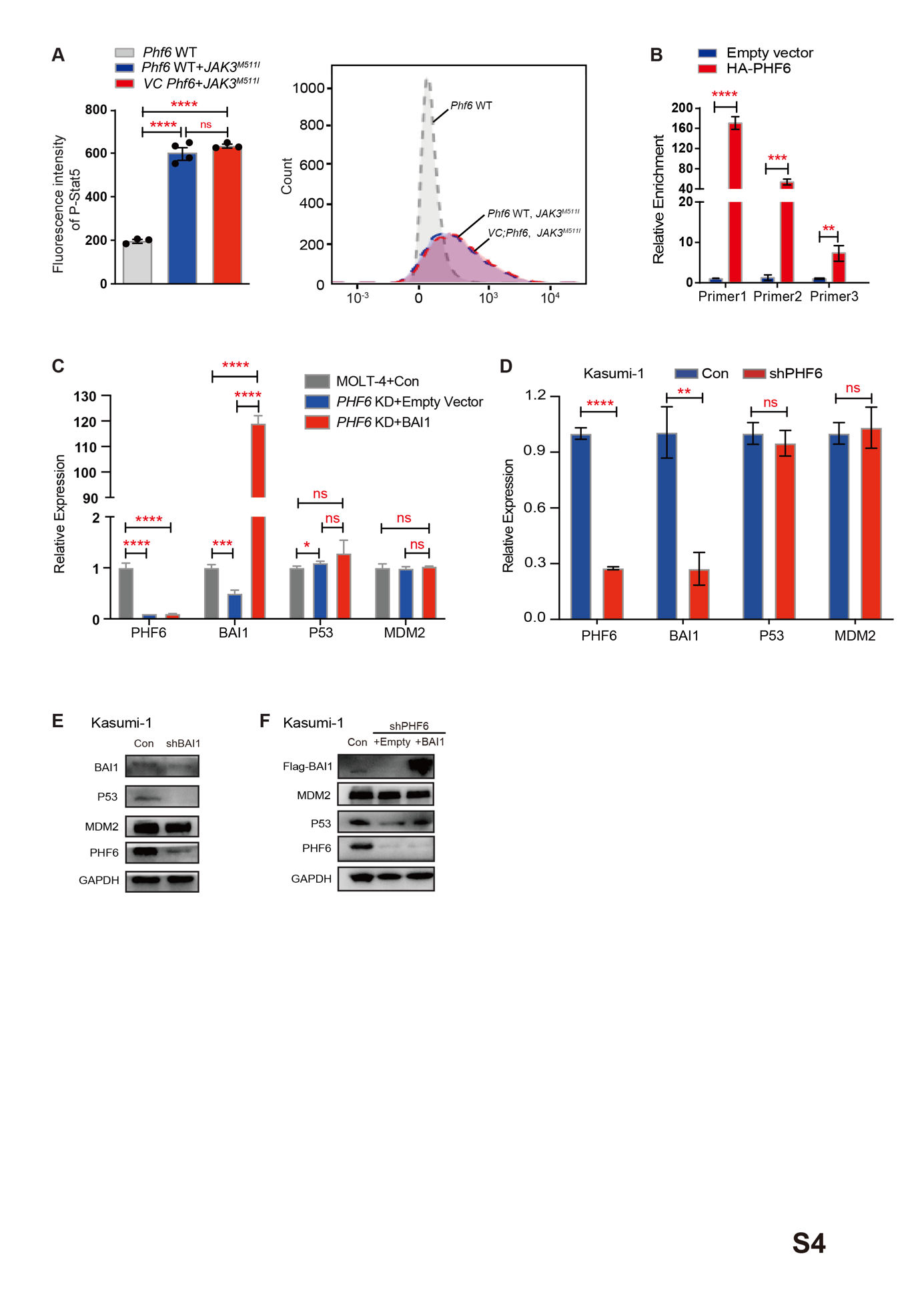
**

**Supplementary Figure 4. Loss of PHF6 decreased BAI1 expression independent of the JAK3-STAT5 signaling pathway.**

(A) The phosphorylation level of Stat5 in *Phf6* WT*+JAK3^M511I^* cells, *VC* *Phf6+JAK3^M511I^* cells and WT cells measured by flow cytometry. (B) HA-PHF6 was enriched by the HA antibody, and ChIP-qPCR was performed to verify the binding of the PHF6 protein to the *ADGRB1* (*BAI1*) DNA sequence in PHF6 OE K562 cells. (C) The mRNA levels of PHF6, BAI1, P53 and MDM2 in PHF6 KD+BAI1 OE MOLT-4 cell, PHF6 KD+Empty Vector MOLT-4 cell and PHF6 Con MOLT-4 cell. (D) The mRNA expression of PHF6, BAI1, P53 and MDM2 in PHF6 KD Kasumi-1 cells and control cells. (E) The protein levels of BAI1, P53, MDM2 and PHF6 in PHF6 KD/Control Kasumi-1 cells. (F) The protein levels of BAI1, P53, MDM2 and PHF6 in PHF6 KD+Flag-BAI1 OE Kasumi-1 cells (lane 3), PHF6 KD+Empty Vector Kasumi-1 cells (lane 2) and PHF6 Con Kasumi-1 cells (lane 1).

**Supplementary Figure 5**

**
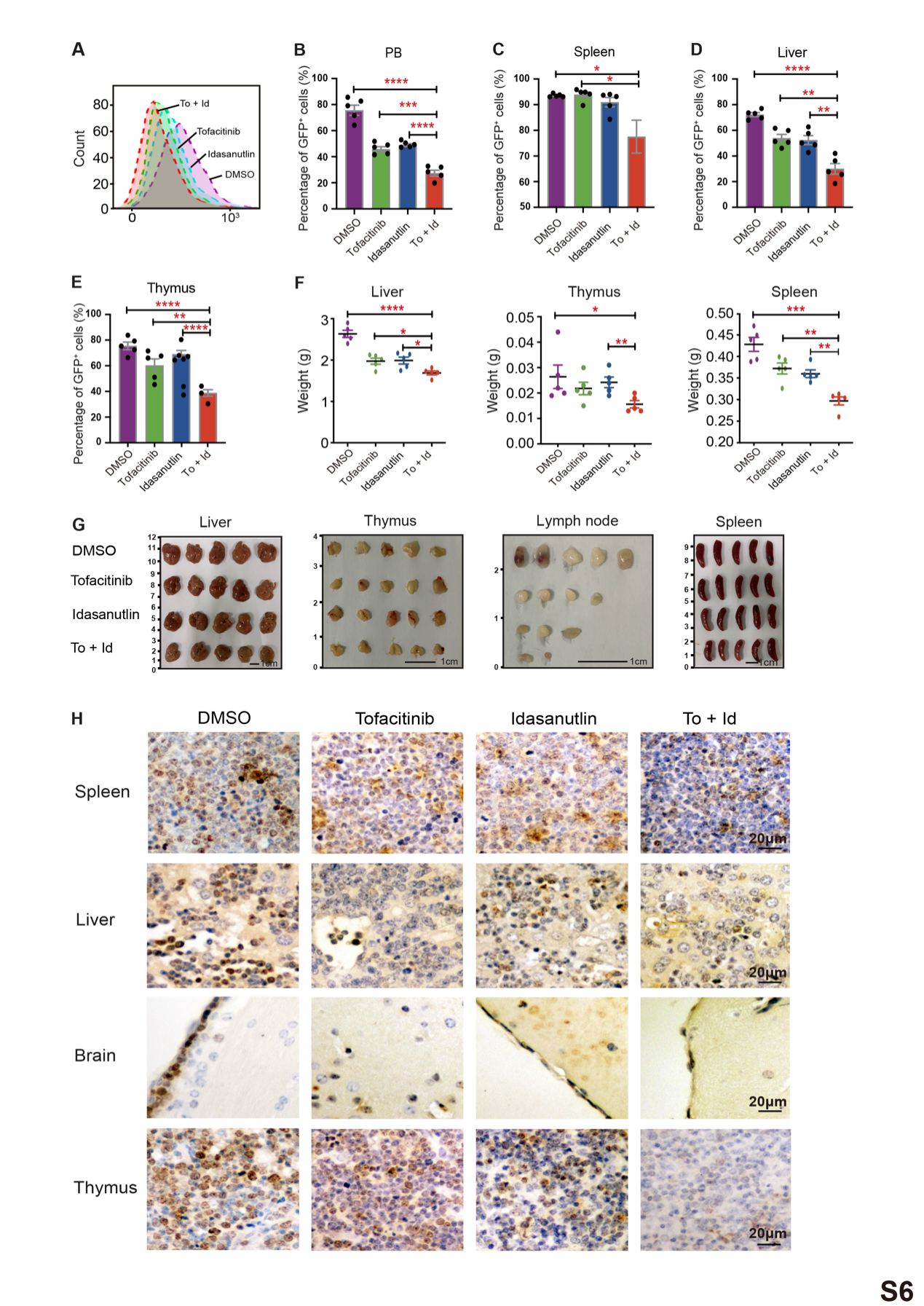
**

**Supplementary Figure 5. Combined treatment with tofacitinib and idasanutlin was effective against T-ALL.**

(A) The phosphorylation level of Stat5 in BM cells from *VC Phf6*+*JAK3^M5111^* T-ALL mice treated with placebo, tofacitinib, idasanutlin, or combined tofacitinib and idasanutlin through oral gavage. (B-E) The percentage of GFP^+^ leukemia cells in PB, spleen, liver and thymus from *VC* *Phf6*+*JAK3^M5111^* T-ALL mice treated with different drugs. (F-G) The weights of liver, thymus and spleen from *VC Phf6*+*JAK3^M5111^* T-ALL mice treated with different drug treatments. (H) Immunohistochemical staining of Ki67 (magnification, 40X) in spleen, liver, brain and thymus from *VC Phf6*+*JAK3^M5111^* T-ALL mice treated with different drugs.

**Supplementary table 1:** The abbreviation list of mouse strains used in this study.

| **Full form** | **Presented form** | **Phf6 WT/KO** |
| --- | --- | --- |
| *Mx1-Cre;Phf6^fl/y^* | *MC Phf6^fl/y^* | WT |
| *Mx1-Cre;Phf6^+/y^* | *MC* | WT |
| *Mx1-Cre;Phf6^fl/y^*+*JAK3^M511I^* | *MC Phf6^fl/y^*+*JAK3^M511I^* | WT |
| *Mx1-Cre;Phf6^+/y^*+*JAK3^M511I^* | *MC*+*JAK3^M511I^* | WT |
| *MC Phf6^fl/y^*+*JAK3^M511I^* injected with pIpC | *MC* *Phf6* KO*+JAK3^M511I^* | KO |
| *MC*+*JAK3^M511I^* injected with pIpC | *MC* *Phf6* WT+*JAK3^M511I^* | WT |
| *Vav1-Cre;Phf6^fl/y^* | *VC* *Phf6* | KO |
| *Phf6^fl/y^* | *Phf6* WT | WT |
| *Vav1-Cre;Phf6^fl/y^*+*JAK3^M511I^* | *VC* *Phf6*+*JAK3^M511I^* | KO |
| *Phf6^fl/y^*+*JAK3^M511I^* | *Phf6* WT+*JAK3^M511I^* | WT |

**Supplementary table 2.** PHF6 and JAK/STAT mutation information of 44 T/M MPAL patients from Alexander *et al*, 2018 [1].

| **SJ Genome ID** | **WHO classification** | **Gender** | **Age (years)** | **Days following diagnosis** | **PHF6 mutation** | **JAK 1-3/STAT mutation** | **JAK3 mutation** |
| --- | --- | --- | --- | --- | --- | --- | --- |
| SJMPAL016447 | T/M | m | 9 | 193 | p. R168fs, frameshift | JAK 1-3/STAT | JAK3 p.M511I, missense |
| SJMPAL016849 | T/M | m | 6 | 74 | p.Y301*, nonsense | JAK 1-3/STAT | JAK3 p.M511I, missense |
| SJMPAL043510 | T/M | f | 10 | 39 | p.R274Q, missense | JAK 1-3/STAT | / |
| SJMPAL003414 | T/M | m | 5 | 20 | p.R116*, nonsense | / |  |
| SJMPAL016340 | T/M | m | 9 | 223 | p.G93fs, frameshift | / |  |
| SJMPAL041121 | T/M | m | 6 | 360 | NA | / |  |
| SJMPAL011914 | T/M | m | 7 | 2813 | p.C17S, missense | / |  |
| SJTALL030047 | T/M | m | 9 | 296 | / | JAK 1-3/STAT | JAK3 p.M511I, missense |
| SJMPAL016108 | T/M | f | 9 | 512 | / | JAK 1-3/STAT | JAK3 p.T848A, missense |
| SJMPAL017976 | T/M | m | 13 | 360 | / | JAK 1-3/STAT | / |
| SJMPAL042791 | T/M | f | 13 | 895 | / | JAK 1-3/STAT | / |
| SJMPAL042794 | T/M | m | 15 | 25 | / | JAK 1-3/STAT | / |
| SJMPAL044945 | T/M | f | 7 | 1089 | / | JAK 1-3/STAT | / |
| SJHM030053 | T/M | m | 17 | 317 | / | JAK 1-3/STAT | / |
| SJMPAL011911 | T/M | m | 15 | 2034 | / | JAK 1-3/STAT | / |
| SJMPAL011913 | T/M | m | 13 | 3406 | / | JAK 1-3/STAT | / |
| SJMPAL011915 | T/M | f | 2 | 198 | / | JAK 1-3/STAT | / |
| SJMPAL012420 | T/M | f | 2 | 4185 | / | JAK 1-3/STAT | / |
| SJMPAL012421 | T/M | f | 12 | 1718 | / | JAK 1-3/STAT | / |
| SJMPAL040027 | T/M | m | 15 | 2590 | / | JAK 1-3/STAT | / |
| SJMPAL040037 | T/M | m | 15 | 963 | / | JAK 1-3/STAT | / |
| SJMPAL040459 | T/M | f | 7 | 892 | / | JAK 1-3/STAT | / |
| SJMPAL042792 | T/M | f | 4 | 180 | / | JAK 1-3/STAT | / |
| SJMPAL042793 | T/M | f | 4 | 546 | / | JAK 1-3/STAT | / |
| SJMPAL042800 | T/M | f | 12 | 364 | / | JAK 1-3/STAT | / |
| SJMPAL011912 | T/M | m | 12 | 3161 | / | JAK 1-3/STAT | / |
| SJMPAL043509 | T/M | m | 8 | 5938 | / | JAK 1-3/STAT | / |
| SJMPAL005001 | T/M | m | 10 | 775 | / | / |  |
| SJMPAL012427 | T/M | f | 16 | 162 | / | / |  |
| SJMPAL016341 | T/M | m | 5 | 1814 | / | / |  |
| SJMPAL016342 | T/M | m | 1 | 295 | / | / |  |
| SJMPAL016343 | T/M | f | 3 | 511 | / | / |  |
| SJMPAL016344 | T/M | f | 5 | 1222 | / | / |  |
| SJMPAL017978 | T/M | f | 2 | 2921 | / | / |  |
| SJMPAL022667 | T/M | m | 16 | 2666 | / | / |  |
| SJMPAL042796 | T/M | m | 3 | 1215 | / | / |  |
| SJMPAL042797 | T/M | f | 6 | 3535 | / | / |  |
| SJMPAL042801 | T/M | m | 5 | 1169 | / | / |  |
| SJMPAL042942 | T/M | m | 3 | 1054 | / | / |  |
| SJMPAL043505 | T/M | m | 3 | 1562 | / | / |  |
| SJMPAL043514 | T/M | f | 4 | 682 | / | / |  |
| SJMPAL043772 | T/M | m | 4 | 771 | / | / |  |
| SJMPAL043773 | T/M | f | 7 | 394 | / | / |  |
| SJMPAL044948 | T/M | m | 1 | 3439 | / | / |  |

**References**

1. Alexander, T.B., et al., *The genetic basis and cell of origin of mixed phenotype acute leukaemia.* Nature, 2018. **562**(7727): p. 373-379.
